# Supplementary material for: A 5 × 200 Gbps microring modulator silicon chip empowered by two-segment Z-shape junctions
Source: Nat Commun. 2024 Jan 31;15:918. doi: 10.1038/s41467-024-45301-3 (PMC10831040; doi:10.1038/s41467-024-45301-3)
Supplement: Supplementary file 1 — Supplementary Information [file 41467_2024_45301_MOESM1_ESM.pdf]

Supplementary Information: A 5×200 Gbps Microring Modulator Silicon Chip Empowered by Two-Segment Z-shape Junctions

Yuan Yuan[1,*], Yiwei Peng[1], Wayne V. Sorin[1], Stanley Cheung[1], Zhihong Huang[1], Di Liang[1], Marco Fiorentino[1], and Raymond G. Beausoleil[1]

[1]Hewlett Packard Labs, Hewlett Packard Enterprise, Milpitas, CA 95035, USA
[*]yuan.yuan@hpe.com

I. Microring waveguide loss

The waveguide loss of the microring can be extracted from the measured transmission spectrum, shown in Fig. S1 (a). The measured finesse of the microring modulator (MRM) is $F = FSR/FWHM$ ~16 and the extinction ratio is $ER \sim$ 16 dB. Both parameters can be expressed using the MRM loss coefficients by[1]

$$F \approx \frac{2\pi}{\delta_\kappa + \delta_r}, \tag{1}$$

$$ER \approx \left|\frac{\delta_\kappa + \delta_r}{\delta_\kappa - \delta_r}\right|^2, \tag{2}$$

where δ_κ is the coupling loss coefficient and δ_r is the microring waveguide propagation loss coefficient. Based on the measured values of F and ER, loss coefficients can be calculated, $\delta_\kappa \sim 0.167$ and $\delta_r \sim 0.229$. The coupling coefficient value is also consistent with the coupler simulation results using Lumerical FDTD. Hence, the transmission spectrum can be plotted by

$$T = \left|\frac{t - ae^{-j\phi}}{1 - tae^{-j\phi}}\right|^2, \tag{3}$$

where t is the field transmission of the coupler, a is roundtrip field transmission of the microring, and ϕ is the roundtrip phase of the microring. The relationship between the field transmission and loss coefficients is $t^2 = e^{-\delta_\kappa}$ and $a^2 = e^{-\delta_r}$. The calculated transmission spectrum according to Eq. 3 is shown in Fig. S1(b), which is in good agreement with the measured transmission spectrum. Since $a^2 = e^{-\delta_r} \sim 0.795$, the actual loss of the MRR is $\sim$ 0.205 (i.e., $\sim$ -1 dB), and the MRR propagation loss per unit length is -133 dB/cm. This value is close to the simulated propagation loss of -129 dB/cm from Lumerical Charge and Mode.

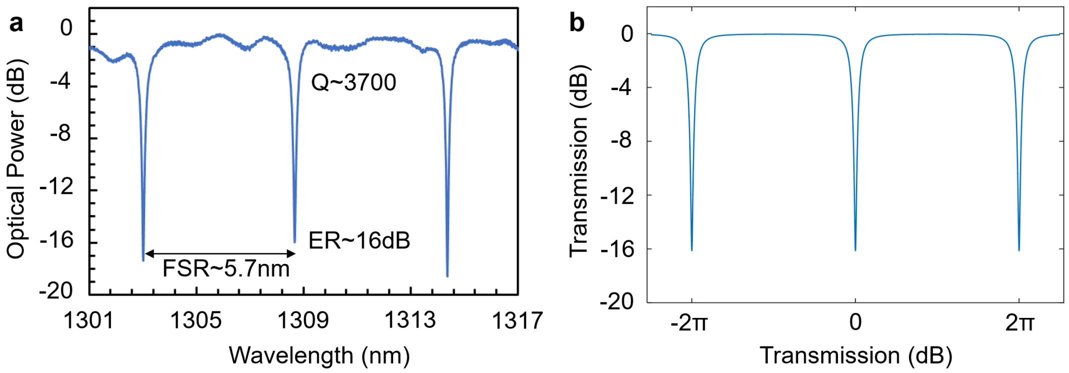

Figure S1. Transmission spectrum. a, Measured and **b**, calculated transmission spectrum of a single channel microring resonator modulator (MRM).

II. Electro-optical response

A Z-shape Si p-n junction has been adopted in the MRM to enhance the overlap between the carrier variance and optical mode. However, the increased depletion interface of the Z-shape junction leads to a larger junction capacitance, which ultimately limits the overall bandwidth of the MRM. To address this trade-off, four dopant implants were applied to the microring waveguide. This design provides an additional degree of freedom to independently adjust the doping concentrations in the slab region and the waveguide core region. Therefore, high modulation efficiency, reasonable free-carrier absorption, and small series resistance can be achieved simultaneously. The small series resistance effectively alleviates the trade-off between modulation efficiency and bandwidth. To attain the next-generation data rate of 200 Gb/s per lane, just reducing the series resistance is insufficient to meet the required electro-optical (EO) bandwidth. Fortunately, the optical digital-to-analog converter (DAC), the two-segment structure, can further improve the resistance-capacitance (RC) bandwidth. Figures 3 (a) and (d) show the measured S11 charts for the least significant bit (LSB) and the most significant bit (MSB) segments at -3 V. The equivalent circuits for both segments can be derived through the S11, shown as insets in Fig. 3 (b) and (e). Although the product of the junction capacitance and series resistance, $C_j \times R_s$, is almost independent of the junction length, the smaller capacitance still provides higher RC bandwidth considering the entire equivalent circuits. The LSB and MSB junctions have RC bandwidths of $\sim$ 79.1 GHz and 64.5 GHz, respectively. As a comparison, the equivalent one-segment MRM would yield a much smaller RC bandwidth of $\sim$ 53.6 GHz.

The measured EO bandwidths of both segments, as depicted in Fig. S2, are close to each other. This is because the RC bandwidths of both segments are higher than the photon lifetime-limited bandwidth $f_{ph} \sim$ 62 GHz. The f_{ph} dominates the EO bandwidth, therefore the overall bandwidth does not vary much. Differently, if the RC bandwidth is less than f_{ph}, it will exhibit a larger influence. For instance, with the one-segment MRM featuring a 53.6 GHz RC bandwidth, the overall EO bandwidth will reduce to $\sim$ 41 GHz. As a result, the two-segment design improves the RC bandwidth by $>$ 20% and the consequent EO bandwidth by $>$ 17%.

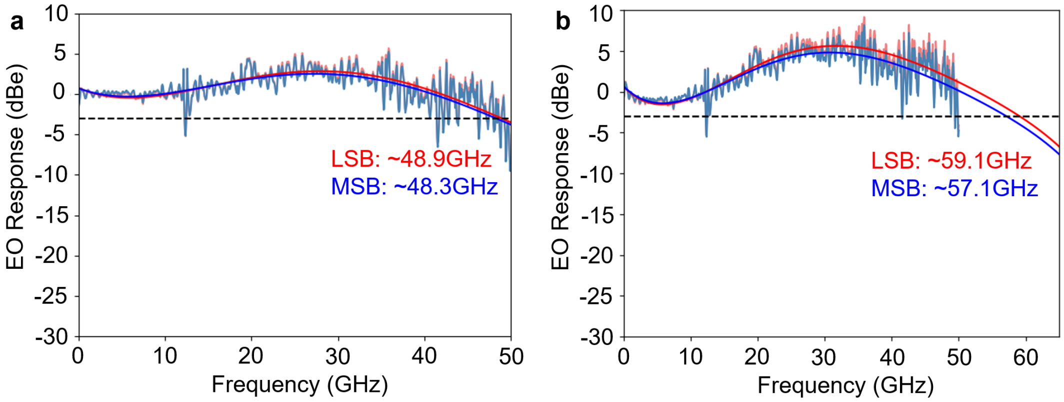

Figure S2. Electro-optical (EO) response. Measured EO response ($|S21|^2$) of the two-segment microring resonator modulator (MRM) at insertion loss of **a**, -6 dB and **b**, -3 dB.

The EO response of the MRM is also affected by wavelength detuning. The MRM can achieve an extended 3 dB bandwidth by tuning the wavelength away from the resonance. Figure S2(a) is the measured EO response of the LSB and MSB segments at the wavelength corresponding to the maximum modulation slope, where the wavelength detuning $\Delta\lambda$ is $\sim$ 0.1 nm. This wavelength is located at the -6 dB insertion loss (IL) point on the transmission spectrum of the MRM. The LSB and MSB have 3 dB bandwidths of $\sim$ 48.9 GHz and 48.3 GHz, respectively. By further detuning the wavelength towards the IL of -3 dB, a distinct optical peaking effect can be observed, as shown in Fig. S2(b). The measured results are limited to 50 GHz due to the bandwidth constraint of the vector network analyzer. The fitted curves indicate that the LSB exhibits an enhanced 3 dB bandwidth of $\sim$ 59.1 GHz and the MSB attains a 3 dB bandwidth of $\sim$ 57.1 GHz.

Thanks to the novel design, this MRM achieves state-of-the-art EO bandwidth with a relatively large radius of 12 μm. By further reducing the MRM radius, the smaller junction capacitance can enhance the EO bandwidth considering the entire equivalent circuit. If the radius reduces to 4 μm, the simulated RC responses of the equivalent circuits of the LSB and MSB are illustrated in Fig. S3 (a) and (b), respectively. As expected, the RC time-limited bandwidth of the LSB extends from $\sim$ 79.1 GHz to $\sim$ 94.3 GHz, and the MSB's RC bandwidth improves from $\sim$ 64.5 GHz to $\sim$ 86.3 GHz. Using the simplified EO bandwidth equation, $f_{est} = f_{RC} f_{ph} / \sqrt{f_{RC}^2 + f_{ph}^2}$, the estimated EO bandwidth of both LSB and MSB will be $>$ 50 GHz without detuning peaking effect, which is comparable to Intel's 4 μm-radius MRM[2].

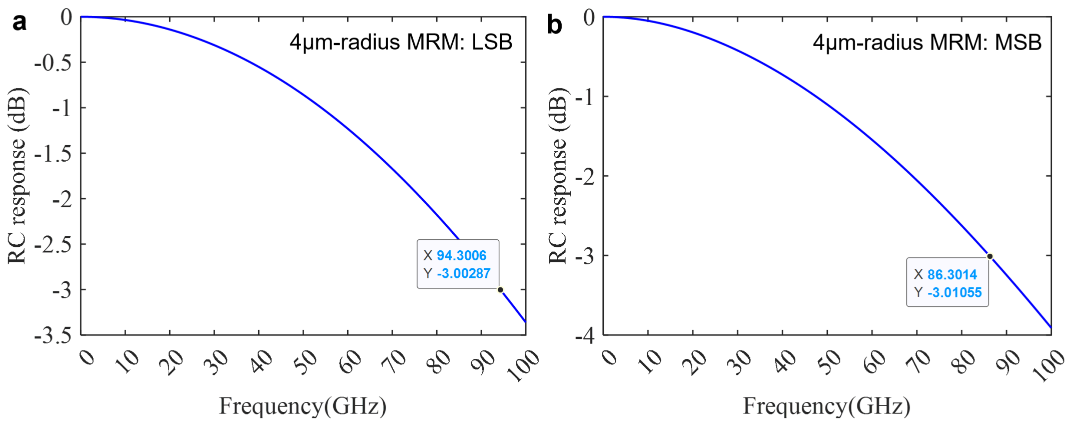

Figure S3. Resistance-capacitance (RC) response. Simulated RC response of the **a**, least significant bit (LSB) and **b**, most significant bit (MSB) of the two-segment microring resonator modulator (MRM) with a 4 μm radius.

IV. Linearity and crosstalk

The segment length ratio of $\sim$ 2:1 makes the MRM an optical DAC to simplify the pulse amplitude modulation with four levels (PAM4) driving signal to two non-return-to-zero (NRZ) driving signals. The equally spaced four levels are critical to realize PAM4 modulation. Figure S4(a) illustrates the measured optical transmission spectrum of the MRM at four bias levels: 1) LSB = 0 V, MSB = 0 V; 2) LSB = -4 V, MSB = 0 V; 3) LSB = 0 V, MSB = -4 V; and 4) LSB = -4 V, MSB = -4V. The resonant wavelength of the MRM red shifts with bias voltage levels. To quantify the linearity of the optical DAC, the measured spectrum on a linear scale is also plotted as shown in Fig. S4(b). At a fixed laser wavelength indicated by the black dash line, the optical power differences of the four bias levels are about 0.16, 0.18, and 0.16, respectively, with a maximum spacing deviation of $\sim$ 8%. This two-segment optical DAC has good linearity and enables nearly equally spaced PAM4 modulation.

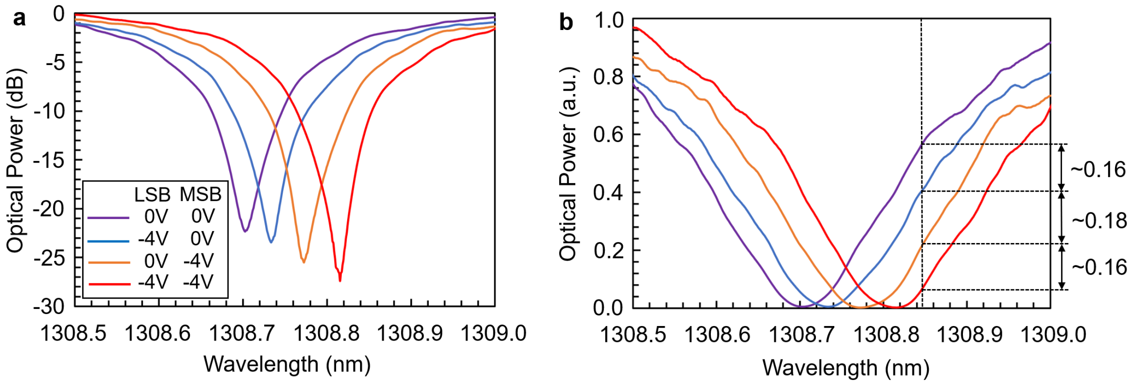

Figure S4. Transmission spectrum versus bias voltage. Measured transmission spectrum of the two-segment microring resonator modulator (MRM) at four bias levels on (a) dB scale and (b) linear scale.

In addition to measuring optical transmission intervals at DC bias voltages, the RF responses of LSB, MSB, and their combination were also conducted to demonstrate the linearity in large-signal RF cases. Figure S5 displays the captured waveforms on the digital communication analyzer (DCA) at 50 Gbaud/s data rate, where the pink waveform indicates driving only LSB, the yellow waveform corresponds to driving only MSB, and the blue curve represents the simultaneous driving of both LSB and MSB. If there is no time offset between the driving signals of LSB and MSB, all three waveforms should exhibit similar shapes. As shown in Fig. S5(a), three curves share similar waveforms. Moreover, the optical modulation amplitude (OMA) ratio of the three waveforms is approximately 1:2:3, underscoring great linearity in the RF domain. It is worth noting that the driving signals and swing voltages are identical for both LSB and MSB. An advantage of the two-segment design is the ability to independently tune the two driving signals, allowing for the possibility of achieving even better linearity by making slight adjustments to the amplitudes of the driving signals. On the other hand, by introducing an integer-bits offset between the driving signals of LSB and MSB, the combined response should exhibit four distinct levels of modulation. Figure S5(b) presents the captured waveforms with a 20 ps offset, i.e., 1 bit, and an example six-bit sequence is depicted by the red dash

lines. The LSB waveform shows a sequence of [0, 1, 1, 0, 1, 0], whereas the MSB waveform is 1 bit later with a sequence of [0, 0, 1, 1, 0, 1]. The combined waveform of both LSB and MSB displays a sequence of [0, 1, 3, 2, 1, 2], aligning with the requirements of the optical DAC. This result not only demonstrates the good linearity of the large-signal RF response but also indicates the absence of noticeable electrical crosstalk.

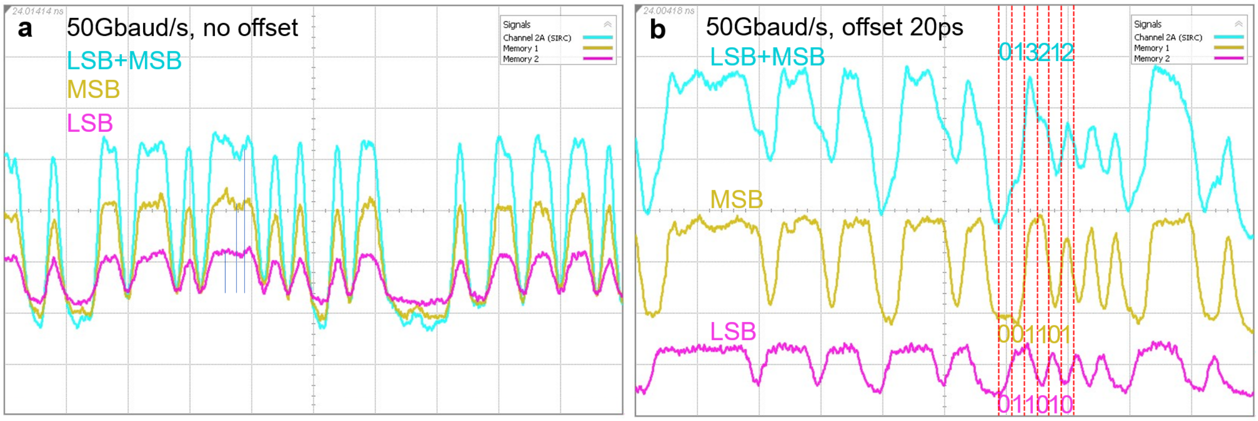

Figure S5. Waveform. Measured waveform of the least significant bit (LSB), most significant bit (MSB), and LSB + MSB at 50 Gbaud/s data rate: **a**, No offset between LSB and MSB drive signals. **b**, 20 ps offset (i.e., 1 bit) between LSB and MSB drive signals.

Due to the limited channel number in the current setup, we can only provide two high-speed driving signals to this MRM array. In order to closely simulate the real product operating condition, which involves driving multiple channels simultaneously using custom complementary metal-oxide-semiconductor (CMOS) driver circuits, we shifted half a channel to evaluate channel crosstalk. The micrograph of the measured MRM array is shown in Fig. S6(a), the customized probe simultaneously probes channel 2 MSB and channel 3 LSB, rather than probing LSB and MSB of one channel. Figure S6(b) and (c) show the measured 100 Gb/s NRZ eye diagrams of the channel 2 MSB. The input laser wavelength is aligned with channel 2, channel 2 MSB driving signal remains on, while the channel 3 LSB driving signal is off (left) and on (right). The signal-to-noise ratios (SNRs) of the two 100 Gb/s NRZ eyes are very close, around 3.1. Likewise, the 100 Gb/s NRZ eye diagrams of the channel 3 LSB are presented in Fig. S6 (d) and (e). Toggling the driving signal off and on of its neighboring channel 2 does not impact the eye quality, both eye diagrams have an SNR of $\sim$ 2.9. Hence, this measurement serves as compelling proof that the dense wavelength division multiplexing (DWDM) MRM array exhibits negligible optical crosstalk under 100 Gbaud/s modulation conditions. Consequently, it is entirely feasible to support a total data rate of 1 Tb/s with this DWDM MRM array.

V. Uniformity

The device uniformity is another important figure of merit. To demonstrate the consistent performance of the Z-shape MRM design, PAM4 eye diagrams were measured across six different dies. As depicted in Fig. S7, all of these MRMs exhibit open 180 Gb/s PAM4 eye diagrams using the same driving signals without device-specific calibration. The demonstration of 180 Gb/s eyes here is because the upper limit of the setup is 200 Gb/s (as detailed in VI. Experimental setup for eye measurement), achieving 200 Gb/s PAM4 eye requires careful calibration and precise tuning of LSB/MSB time delay for each device. The die within the red block is the one measured and reported in the main manuscript, and the six surrounding dies show similar performance. This Z-shape design is realized through four standard implantations rather than the shaded implantations, making it well-suited for mass production. The exceptional uniformity of this design paves the way for the commercialization of high-speed, efficient two-segment Z-shaped MRMs, enabling their widespread deployment.

VI. Experimental setup for eye measurement

The experimental setup for eye diagram measurement is shown in Fig. S8. A dual-channel 120 GSa/s arbitrary waveform generator (AWG) M8194A was used to provide NRZ signals for the LSB and MSB. It can generate frequency content up to 50 GHz. An external signal generator was used to provide 2.5 GHz square wave signals to synchronize the AWG and the DCA. The two NRZ driving signals were amplified by two identical 60 GHz electrical power amplifiers (SHF S804 B), passed through two identical 50 GHz bias-tees (11612B), and provided 1.6 V swing voltages on both LSB and MSB. The driving signals were calibrated using AWG internal calibration function to compensate for the response of the amplifiers, bias-tees, and RF cables.

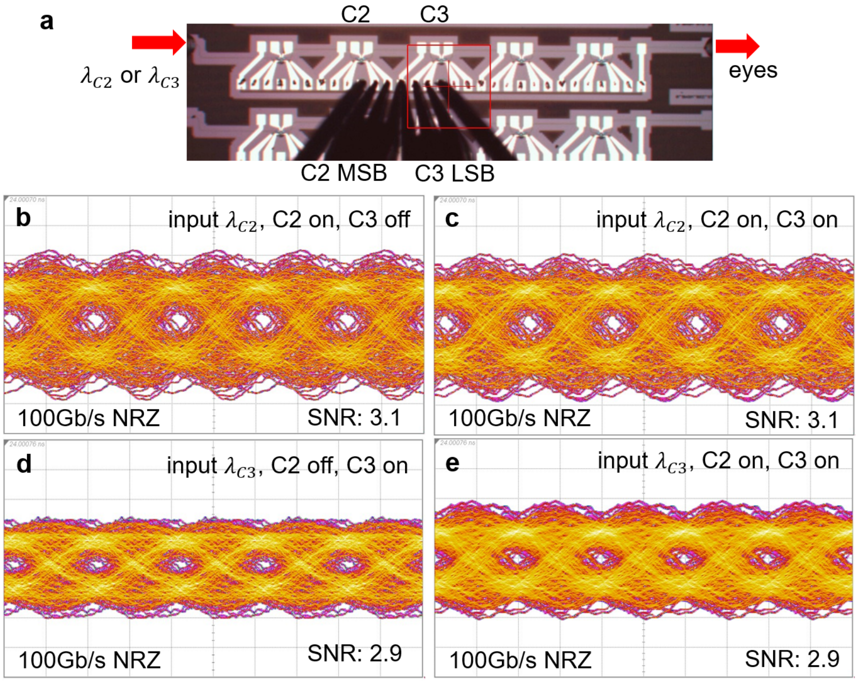

Figure S6. **Channel crosstalk.** **a**, Micrograph of the probed microring resonator modulator (MRM) array for large signal crosstalk measurements. Measured 100 Gb/s NRZ eye diagrams: input wavelength is λ_{C2}, C2 driving signal is on, C3 driving signal is **b**, off and **c**, on; input wavelength is λ_{C3}, C3 driving signal is on, C2 driving signal is **d**, off and **e**, on.

By delaying integer bits between the two driving NRZ signals, the two-segment MRM can generate optical PAM4 signals. A customized probe was used to probe one MRM at a time. The five 200 Gb/s PAM4 eye diagrams were measured channel by channel. Due to the 50 GHz bandwidth of the AWG and bias-tees, 200 Gb/s PAM4 is the upper limit of this experimental setup. By increasing the bandwidth of the setup, the quality of the eye diagrams and the data rate can be further improved.

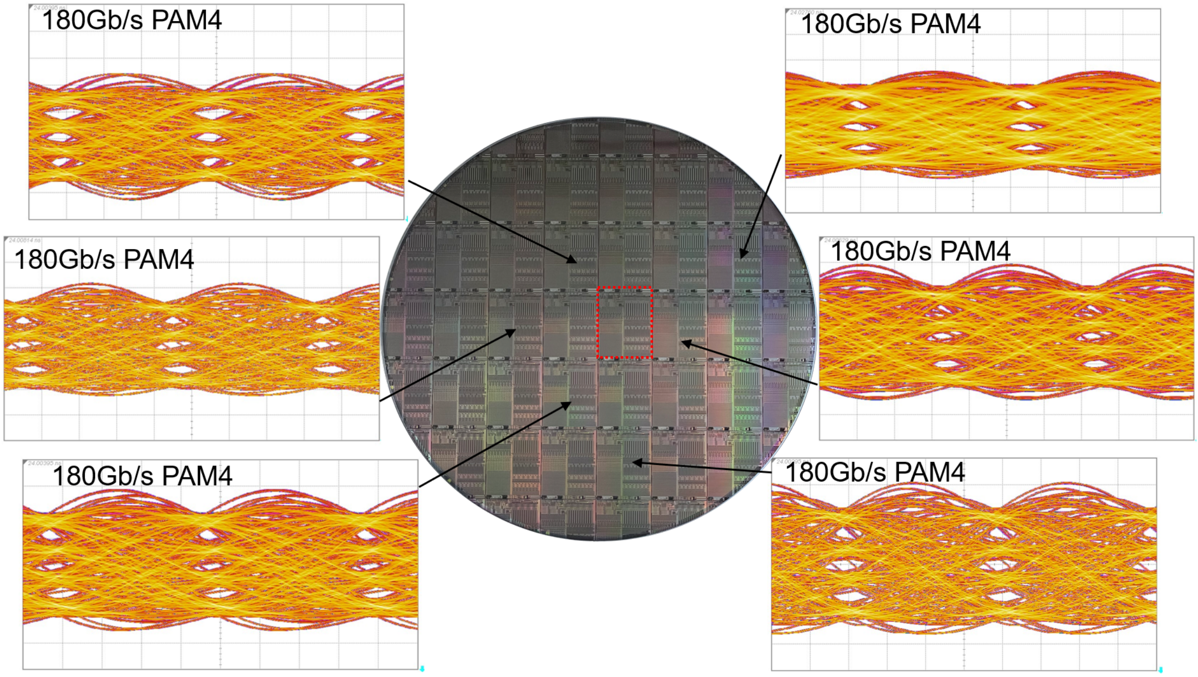

Figure S7. Uniformity. Measured 180 Gb/s PAM4 eye diagrams of the two-segment Z-shape MRMs on different dies.

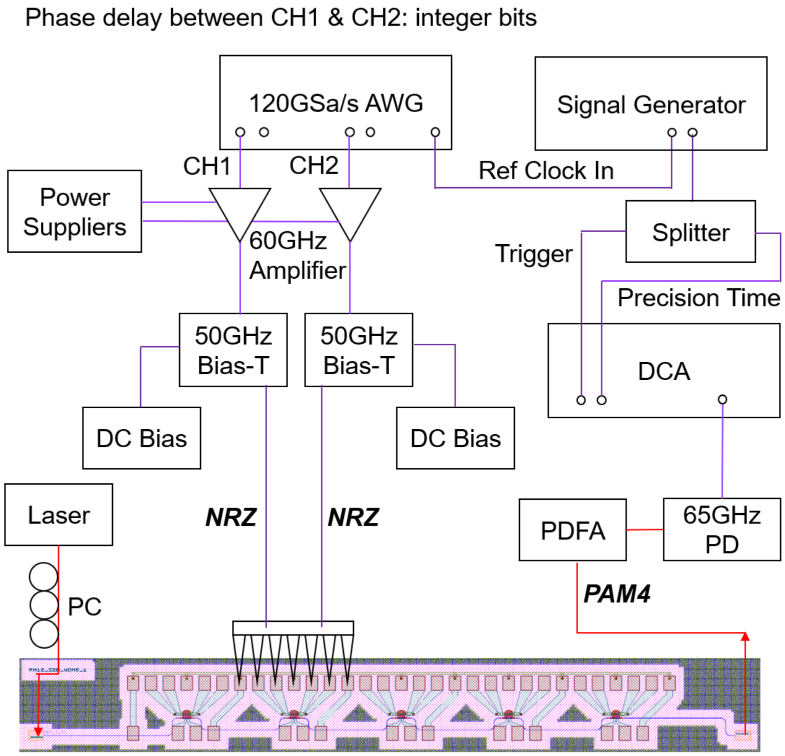

Figure S8. Setup. Experimental setup for eye diagram measurement.

Supplementary References

1. Yuan, Y. *et al.* Mechanisms of enhanced sub-bandgap absorption in high-speed all-silicon avalanche photodiodes. *Photonics Res.* **11**, 337–346 (2023).
2. Sakib, M. *et al.* A 240 gb/s pam4 silicon micro-ring optical modulator. In *2022 Optical Fiber Communications Conference and Exhibition (OFC)*, 01–03 (IEEE, 2022).
